# Non-aneurysmal Gastroepiploic Arterial Hemorrhage With Median Arcuate Ligament Syndrome

**DOI:** 10.7759/cureus.70692

**Published:** 2024-10-02

**Authors:** Hiroki Nagasawa, Youichi Yanagawa

**Affiliations:** 1 Acute Critical Care Medicine, Juntendo University Shizuoka Hospital, Izunokuni, JPN

**Keywords:** celiac axis compression syndrome, defecation strain, gastroepiploic artery, intraperitoneal hemorrhage, laparotomy, median arcuate ligament syndrome

## Abstract

Spontaneous non-aneurysmal gastroepiploic arterial hemorrhage is a rare occurrence, and its association with celiac axis compression syndrome (CACS), also referred to as median arcuate ligament syndrome (MALS), is even more uncommon. Furthermore, nontraumatic intraperitoneal hemorrhage due to defecation strain is also rare. This study reports an extremely rare case of non-aneurysmal gastroepiploic arterial hemorrhage with CACS/MALS after defecation strain. A 24-year-old man presented with a sudden upper abdominal pain on the left side after defecation. The patient was diagnosed with bleeding from the gastroepiploic artery and CACS/MALS using contrast-enhanced computed tomography. The patient underwent urgent laparotomy, and subsequent pathogenic examination revealed no aneurysm. This was an atypical case of intraperitoneal hemorrhage with CACS/MALS, and hemorrhage may have occurred due to a combination of vascular fragility, elevated arterial blood pressure, and hemostatic disorder.

## Introduction

Non-traumatic intraperitoneal arterial hemorrhage is a rare and life-threatening condition often caused by the rupture of an aneurysm [1]. Celiac axis compression syndrome (CACS) is an extraluminal compression of the celiac artery/axis by the median arcuate ligament of the diaphragm. Celiac axis compression syndrome is also referred to as median arcuate ligament syndrome (MALS), and, though uncommon, it may potentially lead to the formation of intra-abdominal arterial aneurysms [2, 3]. Notably, 3%-18% of CACS/MALS cases involve pancreaticoduodenal artery aneurysms [2]. However, aneurysm in the gastroepiploic artery with CACS/MALS is rare and has only been reported once [4]. In this study, we report an extremely rare case of non-aneurysmal gastroepiploic arterial hemorrhage with CACS/MALS due to defecation strain. Non-traumatic intraperitoneal hemorrhage is not well known and also lacks clear evidence. In the present study, we also reviewed the literature on three factors, namely vascular fragility, elevated arterial blood pressure, and hemostatic disorder, that could contribute to idiopathic intraperitoneal hemorrhage.

## Case presentation

**Figure 1:**
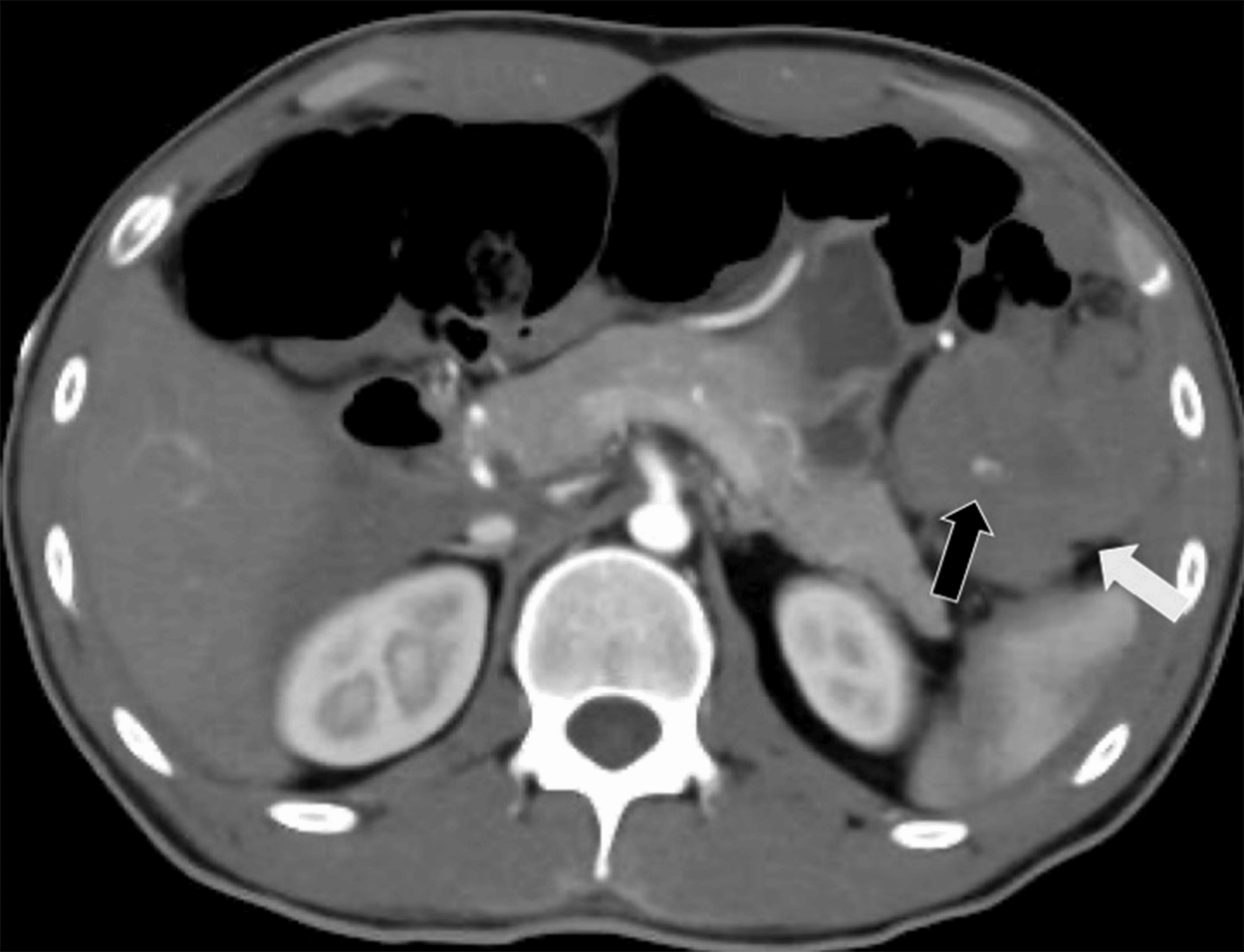
Contrast-enhanced computed tomography reveals intraperitoneal hemorrhage The white arrow indicates a clot around the omentum. The black arrow indicates the extravasation of the contrast media.

A 24-year-old man weighing 58 kg and 175 cm in height presented to our emergency department with sudden upper abdominal pain on the left side for > six hours. The patient was diagnosed with polyarteritis nodosa four years ago and was taking daily medication consisting of 10 mg methylprednisolone, aspirin, methotrexate, ciclosporin, sulfamethoxazole/trimethoprim (infection prophylaxis of steroid use), folic acid, atorvastatin, febuxostat, beraprost sodium, and loxoprofen. The patient denied any history of trauma and reported that abdominal pain occurred after defecation. His vital signs recorded at a previous clinic were as follows: alert consciousness, heart rate 113 beats/min, blood pressure 92/50 mmHg, and respiratory rate 32 breaths/min. Following the administration of 500 mL of Ringer’s solution at the clinic, his vital signs were stable upon arrival to our hospital, as follows: heart rates 90 beats/min, blood pressure 110/64 mmHg, and respiratory rate 20 breaths/min. Abdominal ultrasonography revealed intraperitoneal fluid around the liver, with poor visibility around the spleen and the point of pain. Laboratory analysis of a blood sample revealed a low hemoglobin level of 10.3 g/dL (standard value: 12.0-15.6 g/dL) and a fibrinogen level of 121 mg/dL (reference value: 150-250 mg/dL), with no evidence of coagulopathy. Contrast-enhanced computed tomography (CT) confirmed the presence of hemoperitoneum without free air and hyperdense focus, indicating extravasated contrast within the hematoma around the omentum (Figure 1).

**Figure 2:**
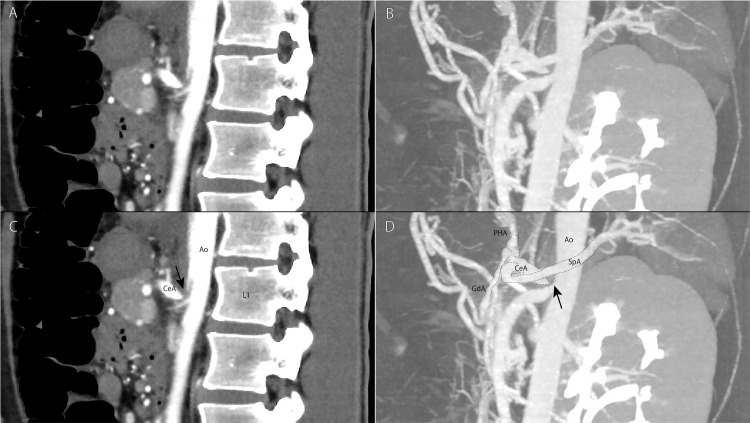
Computed tomography images of the median arcuate ligament Sagittal view (A and C) and 3D re-structured (B and D) contrast-enhanced computed tomography images show the presence of median arcuate ligament syndrome. The black arrow indicates narrowing of the celiac artery. A characteristic hooked appearance is observed owing to the proximal narrowing of the celiac artery with inferior displacement due to indentation by the median arcuate ligament. Ao: aorta; CeA: celiac artery; L: lumber vertebra; GdA: gastroduodenal artery; PHA: proper hepatic artery; SMA: superior mesenteric artery; SpA: splenic artery

Additionally, the CT scan revealed a narrowing at the origin of the celiac artery (Figure 2).

Transarterial embolization (TAE) was not an option in this case because of CACS/MALS. Therefore, the patient underwent an urgent laparotomy. An upper midline incision was made, which revealed approximately 1000 mL of intraperitoneal blood and omentum, including a large hematoma. We performed a partial omentectomy using LigaSure^TM^ (Medtronic, Minneapolis, MN), and subsequent histopathological examination revealed no evidence of aneurysm, malignancy, or vasculitis. The patient was diagnosed with idiopathic left gastroepiploic arterial hemorrhage on intraoperative findings and macroscopic examination of pathology specimens and was kept under observation in the intensive care unit until hospitalization day two and was discharged on hospitalization day 11. No blood transfusion was required during hospitalization.

## Discussion

Celiac axis compression syndrome/MALS is more prevalent in females with low weight aged 30-50 years. It is characterized by postprandial abdominal pain, weight loss, nausea, and vomiting; however, most patients with CACS/MALS do not experience any clinical issues. Radiological investigations using Doppler ultrasonography, CT angiography, or magnetic resonance angiography often reveal celiac artery stenosis with post-stenotic dilation, generally exacerbated by expiration [5]. In the present case study, the examination revealed two interesting aspects. First, the bleeding in the patient originated from the gastroepiploic artery, which is not a typical site of bleeding associated with CACS/MALS. Second, aneurysm rupture, which is commonly observed in patients with CACS/MALS-related bleeding, was not observed in the present case. To the best of our knowledge, non-aneurysmal idiopathic spontaneous gastroepiploic arterial hemorrhage with CACS/MALS has not been reported previously.

To date, only one case of bleeding from a gastroepiploic arterial aneurysm with CACS/MALS has been reported [4]. Consequently, the relationship between gastroepiploic arterial aneurysms and CACS/MALS is not well understood owing to the limited number of cases and lack of clear evidence. Bleeding originates from the pancreaticoduodenal arcades in most cases of intra- or retroperitoneal hemorrhage with MALS [2] because of compression of the celiac artery due to CACS/MALS, which increases the retrograde flow volume of the pancreaticoduodenal arcade, leading to aneurysm formation [6]. Blood flow in the gastroepiploic artery may increase due to CACS/MALS, depending on the position or angle of the branch of the right gastroepiploic artery from the gastroduodenal artery. In the present case, it was unclear whether there were any abnormalities in the branches of the gastroepiploic artery due to vasospasm.

**Figure 3:**
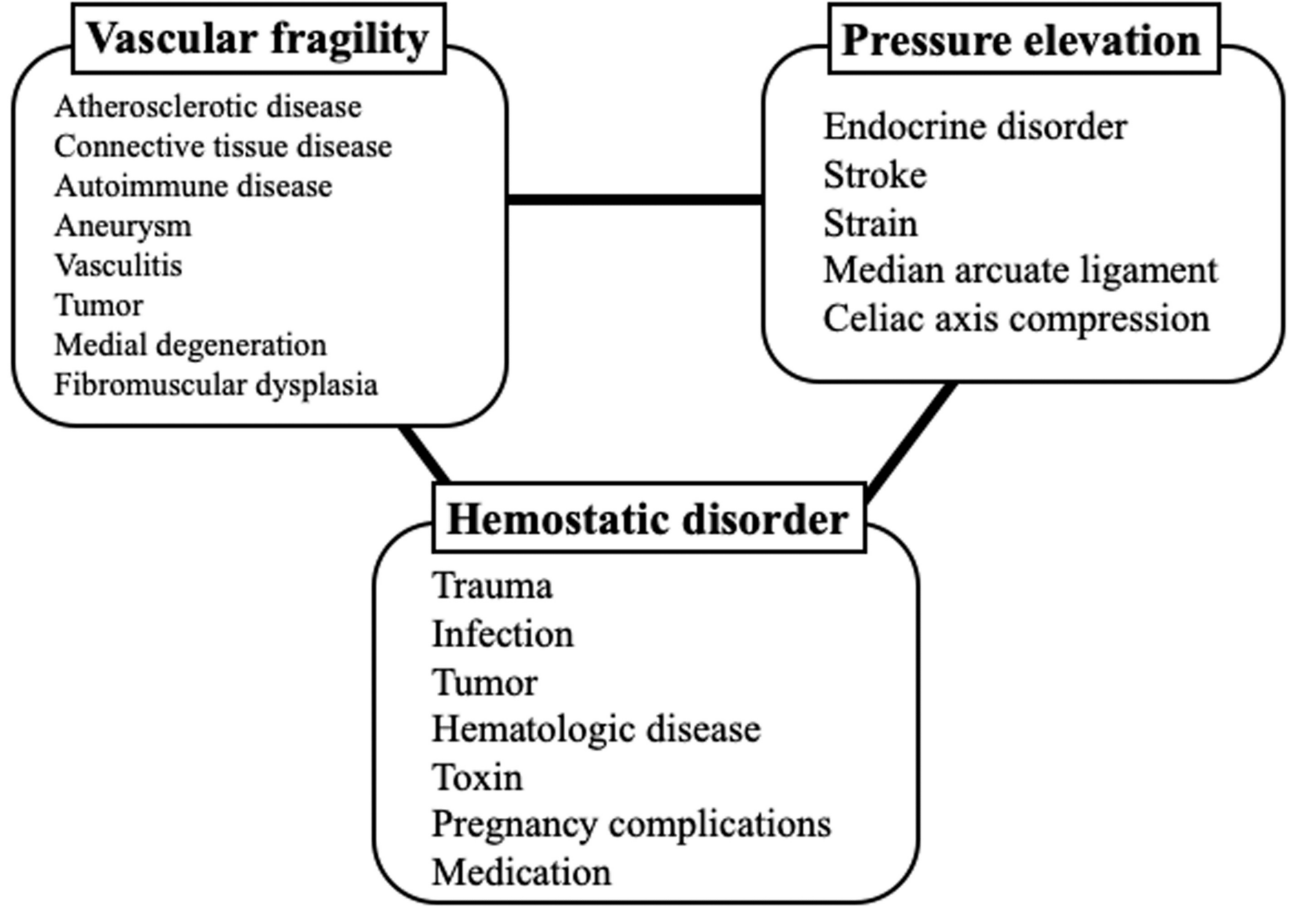
Factors associated with spontaneous intraperitoneal hemorrhage Image credits: Hiroki Nagasawa

The present case was diagnosed as idiopathic spontaneous gastroepiploic arterial hemorrhage without evidence of trauma, (pseudo-) aneurysm, tumor, fibromuscular dysplasia, arterial mediolysis, or vasculitis. We believe that vascular fragility, elevated arterial blood pressure, and hemostatic disorder may have combinedly contributed to the idiopathic spontaneous intraperitoneal hemorrhage (Figure 3).

Vascular fragility is a structural abnormality resulting from conditions such as atherosclerotic diseases, connective tissue diseases, autoimmune diseases, aneurysms, vasculitis, tumors, medial degeneration, and fibromuscular dysplasia [1, 7-12]. The patient in our case had polyarteritis nodosa and was receiving chronic steroid therapy. Arterial blood pressure can increase due to endogenous factors, such as endocrine disorders, stroke, or strain [13-15], or structural issues such as CACS/MALS syndrome or celiac axis compression [6]. The patient had CACS/MALS and experienced defecation strain prior to the onset of abdominal pain. Furthermore, trauma, infection, tumors, hematologic diseases, toxins, pregnancy complications, or medication can cause hemostatic disorders [16-19]. In the present case, the patient had a history of aspirin therapy (100 mg/day). Notably, the interplay between these three factors may have induced intraperitoneal hemorrhage. Consequently, the patient experienced idiopathic spontaneous intraperitoneal hemorrhage.

However, the optimal treatment for ruptured gastroepiploic arteries remains controversial, and both laparotomy [10, 11, 20] and TAE [1, 4, 21] have been successfully performed. In one case involving MALS [4], TAE of the ruptured right gastroepiploic artery was performed via the superior mesenteric artery. In contrast, we chose an urgent laparotomy because of the following: First, whether the gastroepiploic artery was the main source of intraperitoneal bleeding was uncertain based on contrast-enhanced CT. Second, surgical intervention appeared more feasible because of the large clots around the omentum. Consequently, our patient had bleeding from the left gastroepiploic artery with vasospasm, which would have been more difficult to access through catheterization via the superior mesenteric artery or a narrowing celiac artery.

## Conclusions

Non-aneurysmal idiopathic spontaneous gastroepiploic arterial hemorrhage is rare, and its association with median arcuate ligament syndrome is even rarer. Consequently, the optimal treatment for this condition remains controversial. The present case highlights the importance of considering the occurrence of CACS/MALS when selecting a treatment approach. Nevertheless, further studies are warranted to establish the most appropriate management strategy for this rare condition.
